# The Infratrigeminal Suprafloccular Approach to Intrapontine Lesions: An Anatomical Overview and Relevance for the Approach to Intrapontine Lesions

**DOI:** 10.7759/cureus.45708

**Published:** 2023-09-21

**Authors:** Alex Roman, Eduardo Anzolin, Larissa Bianchini

**Affiliations:** 1 Neurological Surgery, Instituto de Neurocirurgia e Cirurgia de Coluna, Passo Fundo, BRA; 2 Neurosurgery Department, Hospital Cristo Redentor, Porto Alegre, BRA; 3 Intensive Care Unit, Universidade de São Paulo, São Paulo, BRA

**Keywords:** safe entry zone, cavernous malformation, microsurgical anatomy, brainstem tumors, intrapontine lesions

## Abstract

Background and objectives

Brainstem lesions have long been considered complex pathologies that may lead to permanent deficits or life-threatening complications, posing significant challenges for surgical removal. Among these lesions, intrapontine lesions are particularly challenging in the field of neurosurgery. However, with advancements in microsurgical anatomy knowledge and technology, these lesions have become more amenable to surgical treatment. In this study, the authors examine an infratrigeminal suprafloccular approach, which has been shown to be a safe surgical route, resulting in fewer postoperative complications, while evaluating the anatomical nuances of the approach and route.

Methods

Twenty cadaveric brainstem specimens were analyzed to assess the anatomy, focusing on the lateral aspect of the pons as a potential safe entry zone for intrapontine lesions. The authors consistently analyzed twenty brainstem specimens, carefully examining the pontine microsurgical anatomy. A triangular area of entrance was measured, with three sides or walls (X, Y, and Z) aiming to identify the safe zone that would spare the distinct pontine nuclei, ascending sensory pathways, corticospinal, corticonuclear, and corticopontine tracts of the brainstem. An illustrative case was adapted to the described safe entry zone for corroboration purposes.

Results

The authors measured three distinct lines on the lateral surface of the pons, named X, Y, and Z, forming a triangle in shape. Line X extended from the midpoint anteroposteriorly of the flocculus of the cerebellum to the apparent trigeminal exit in the lateral aspect of the pons. Line Y ran from the trigeminal exit in the pons to the apparent exit of the facial-vestibulocochlear complex in the far lateral aspect of the pontomedullary sulcus in the cerebellopontine fissure. Line Z represented the measurement from the vestibulocochlear complex to the midpoint anteroposteriorly of the flocculus of the cerebellum. The mean measurements were as follows: X = 14.41mm (range: 10mm to 20mm), Y = 13.1mm (range: 10mm to 21mm), and Z = 3mm (range: 2mm to 5mm). The mean surface area of the analyzed specimens within the triangle (formed by X, Y, and Z) was 20.1mm² (range: 10mm² to 40mm²). This area was identified as a safe zone for the entry of microsurgical approaches to intrapontine lesions, involving less retraction of the anterior pons and potentially sparing critical structures, such as the corticospinal tracts, pontine perforating arteries, tegmentum pontis, cranial nerve nuclei, substantia reticulata dorsally, and transverse pontine fibers. Microsurgical anatomical findings, combined with intraoperative monitoring in an illustrative case, consistently demonstrated that this entry area predicted less functional instability of the analyzed tracts and resulted in fewer postoperative complications.

Conclusion

Deep-seated pontine lesions present a complex range of pathologies with a high potential for devastating outcomes, particularly those involving hemorrhage. This study identifies and describes a presumed safe entry zone that allows for the creation of a surgical corridor for biopsy or microsurgical resection of these lesions, reducing morbidity in a previously considered impenetrable region.

## Introduction

Brainstem lesions present a formidable challenge in neurosurgery due to their intricate location and potential impact on vital neural structures. Among these lesions, intrapontine lesions are particularly complex and have historically posed significant risks during surgical removal. These challenges arise from the brainstem's compact anatomy, which contains crucial nuclei, pathways, and cranial nerve structures that are essential for preserved neurological function. Consequently, surgical intervention in this region requires precision and careful consideration to achieve favorable outcomes [1].

Recent advances in microsurgical techniques and anatomical knowledge have provided new insights into safe entry zones to the brainstem, offering potential avenues for effective surgical management. Notably, Cavalcanti et al. (2015) extensively investigated microsurgical anatomy to identify these safe entry zones [1]. They elucidated crucial areas that allow access to brainstem lesions while minimizing the risk of damage to critical neural elements.

For brainstem lesions, stereotactic biopsy has been considered the "golden standard" for establishing diagnoses [2]. This less invasive approach aids in obtaining essential diagnostic information without significant surgical manipulation, making it an invaluable tool in guiding treatment decisions. On another note, the nature of adult brainstem gliomas has been explored by Reyes-Botero et al., emphasizing the complexities and challenges associated with managing these tumors [3]. Their study sheds light on the importance of tailored treatment strategies based on the tumor's location and characteristics.

Cavernous malformations located in the brainstem represent another subset of lesions requiring specialized attention [1]. Porter et al. [4] shared their experience with 100 patients harboring brainstem cavernous malformations, highlighting the intricacies and unique considerations involved in their management. In the context of cavernous malformations, Recalde et al. investigated the microsurgical anatomy of the safe entry zones on the anterolateral brainstem [5]. Their findings provided valuable information for surgical approaches to cavernous malformations, aiming to minimize surgical risk while optimizing lesion removal.

In light of the challenges posed by intrapontine lesions, this study seeks to build upon the existing research, such as the study by Cavalcanti et al. [1], to further investigate microsurgical anatomy and surgical nuances for intrapontine lesion resection. By examining specific safe entry zones and their relationship to critical neural structures, this research aims to enhance our understanding of surgical interventions in this frangible region. The ultimate goal is to improve patient outcomes and reduce morbidity associated with intrapontine lesion resection, thereby advancing the field of neurosurgery and benefiting patients with these challenging brainstem pathologies.

## Materials and methods

To analyze the superficial anatomy of the brainstem in different cadaveric specimens, the authors measured the mean entry point in the lateral aspect of the pons. Twenty distinct glycerine cadaveric brainstem specimens were utilized in this study to identify a safe zone that would spare the distinct pontine nuclei as well as the ascending and descending pathways while preserving the integrity of the corticospinal, corticonuclear, and corticopontine tracts. The objective is to minimize manipulation of the pontine parenchyma during surgical intervention. The study was conducted at Hospital São Vicente de Paulo & Universidade de Passo Fundo, Passo Fundo, Brazil.

**Figure 1:**
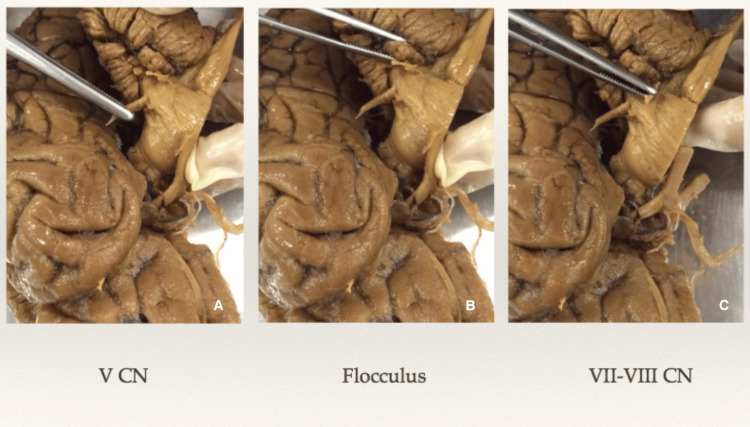
Landmarks at the lateral surface of the pons A: trigeminal nerve exiting point; B: flocculus of the cerebellum; C: facial-vestibulocochlear nerve complex exiting point

A comprehensive understanding of the posterior fossa and brainstem anatomy, particularly the pontine structures, is crucial for developing various approaches to accessing intrapontine lesions. In this study, twenty cadaveric brainstem specimens were meticulously examined to identify potential landmarks for an entry zone for intrapontine lesions (Figure 1). These landmarks included the trigeminal cranial nerve, the cerebellar flocculus, and the facial/vestibulocochlear cranial nerve complex.

By connecting these three identified landmarks, a triangular entry area was observed and designated by its three sides, referred to as X, Y, and Z for analytical purposes. The measurements of these three sides, along with the height of the triangle in the lateral aspect of the pons, were undertaken in millimeters utilizing anatomy laboratory measuring rulers. Additionally, the area of the triangular zone was calculated in mm^2^ to describe the entry area for intrapontine lesions, precisely correlating to an area anterior to the middle cerebellar peduncle, which would be amenable to manipulation during surgical interventions [6].

To ensure accurate measurements without the significant volume modifications typical of in vivo intraoperative findings, glycerine cadaveric brainstem specimens were employed, which maintain more of the normal human anatomical characteristics. This approach allowed for a more stable and reliable comparison of anatomical landmarks, including the trigeminal cranial nerve, cerebellar flocculus, and facial and vestibulocochlear cranial nerve complexes.

By employing these standardized measurement techniques and utilizing glycerine cadaveric brainstem specimens, the authors sought to elucidate a safe and precise entry zone for intrapontine lesions. The results of this study are anticipated to provide valuable insights into surgical planning and techniques, aiming to enhance patient outcomes and minimize potential complications during and after the resection of intrapontine lesions.

## Results

Safe entry zones of the brainstem, particularly the pons, have been extensively studied and reported in the literature due to their complex anatomical characteristics and potential for catastrophic outcomes. Detailed descriptions of the anatomic safe entry zones, including different entry points of the midbrain, medulla, and pons, have been previously established [7-11].

**Figure 2:**
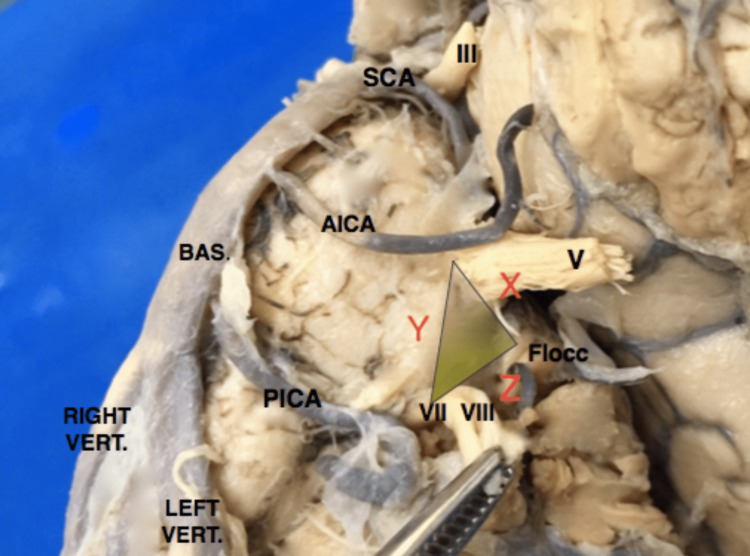
Lateral pons triangle Lateral pons triangle formed by X, Y, and Z lines from the aforementioned landmarks and the area surface of the triangle in yellow shade

In this study, the brainstem specimens were analyzed with a specific focus on the pontine microsurgical anatomy. Based on microsurgical findings, we measured three lines (X, Y, and Z), forming a triangular configuration for didactic purposes (Figure 2). Line X extended from the midpoint of the flocculus of the cerebellum anteroposteriorly to the trigeminal apparent exit in the lateral aspect of the pons. Line Y extended from the trigeminal exit point to the apparent exit point of the facial-vestibulocochlear complex in the far lateral aspect of the pontomedullary sulcus in the cerebellopontine fissure. Line Z represented the measurement from the vestibulocochlear complex to the flocculus of the cerebellum.

The mean measurements for the studied specimens were as follows: X was 14.41 mm (ranging from 10 to 20 mm; SD: 2.91 mm), Y was 13.1 mm (ranging from 10 to 21 mm; SD: 3.03 mm), and Z was 3 mm (ranging from 2 to 5 mm; SD: 1.21 mm), resulting in a mean surface area of 20.1 mm^2^ (ranging from 10 to 40 mm2; SD: 9.38 mm^2^) in the analyzed specimens (Table 1).

**Table 1:** Specimens’ measurements of the infratrigeminal triangle

	NC V - NC VIII (Y)	NC V - Flocculus (X)	NC VIII - Flocculus (Z)	Area
Specimen 1	12 mm	10 mm	3 mm	18 mm^2^
Specimen 2	10 mm	13 mm	4 mm	20 mm^2^
Specimen 3	10 mm	12 mm	2 mm	10 mm^2^
Specimen 4	13 mm	15 mm	2 mm	11 mm^2^
Specimen 5	15 mm	14 mm	4 mm	26 mm^2^
Specimen 6	18 mm	14 mm	2 mm	18 mm2
Specimen 7	13 mm	11 mm	3 mm	18 mm^2^
Specimen 8	18 mm	20 mm	2 mm	16 mm^2^
Specimen 9	21 mm	20 mm	5 mm	40 mm^2^
Specimen 10	15 mm	12 mm	3 mm	19.5 mm^2^
Specimen 11	13 mm	16 mm	4 mm	30 mm^2^
Specimen 12	12 mm	16 mm	2 mm	15 mm^2^
Specimen 13	17.5 mm	20 mm	3.5 mm	31.5 mm^2^
Specimen 14	13.5 mm	15 mm	2.5 mm	19.3 mm^2^
Specimen 15	10 mm	13 mm	1.5 mm	9.75 mm^2^
Specimen 16	13 mm	17 mm	4.5 mm	36 mm^2^
Specimen 17	11 mm	16 mm	3 mm	21 mm^2^
Specimen 18	13 mm	16 mm	2 mm	15 mm^2^
Specimen 19	14 mm	17 mm	1.5 mm	12 mm^2^
Specimen 20	10 mm	14 mm	1 mm	6,5 mm^2^
Median	13.6 mm	15.1 mm	2.275 mm	19.62 mm^2^
SD (Standard Deviation)	3.03 mm	2.91 mm	1.21 mm	9.38 mm^2^
SE (Standard Error)	0.678 mm	0.651 mm	0.271 mm	2.098 mm^2^

**Figure 3:**
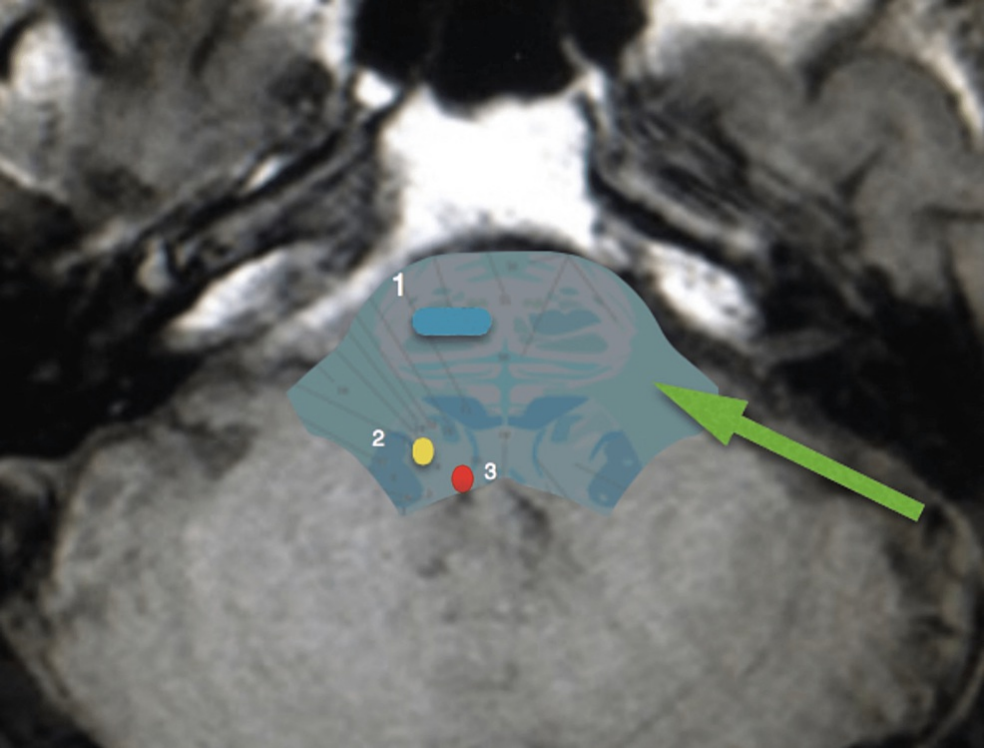
Transvere slice through midpons Midpons slice 1: anteriorly placed descending fibers; 2: facial nerve nucleus; 3: abducens nerve nucleus The green arrow shows the retrosigmoid access location to the lateral surface of the pons

The triangular surface formed by X, Y, and Z, called herein the infratrigeminal triangle or trigone, represented a safe entry zone on the lateral aspect of the pons for microsurgical approaches to intrapontine lesions. This zone was situated immediately anterior to the middle cerebellar peduncle, an area that would usually not include the bulk of superior, inferior, or medial cerebellar peduncle fibers, preserving the anteriorly placed descending fibers of the corticospinal tracts as well as ascending fibers from the posterior columns of the spinal cord and inferior brainstem. The triangular surface between the trigeminal nerve and vestibulocochlear nerve exiting zones and the flocculus of the cerebellum seems to preserve the superficial functional structures as well as deeper functional tissue within the trajectory of deep-seated lesions. When utilizing this route for surgical resection of pontine lesions, we observed less retraction of the anterior pons, which spared crucial anatomical structures such as the corticospinal descending tracts and pontine perforating arteries located ventrally in the pons, and the tegmentum pontis, containing cranial nerve nuclei and the substantia reticulata, dorsally (Figure 3).

**Figure 4:**
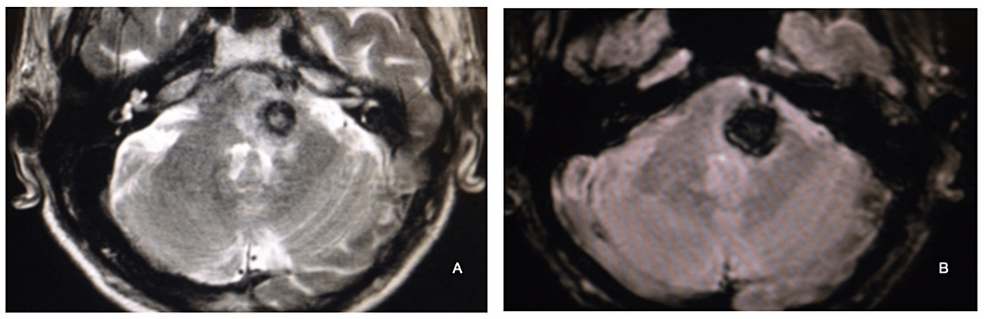
Left intrapontine lesion, consistent with a ruptured cavernous malformation A left pontine ruptured cavernous malformation on a 67-year-old male patient, presenting with right-side hemiparesis, left-side abducens, and facial palsy. A: T2WI MRI with a left pontine heterogenous image is typical of a ruptured cavernous malformation; B: SWI MRI showed evidence of a hypointense lesion, consistent with the hemorragic component of a ruptured cavernous malformation

**Figure 5:**
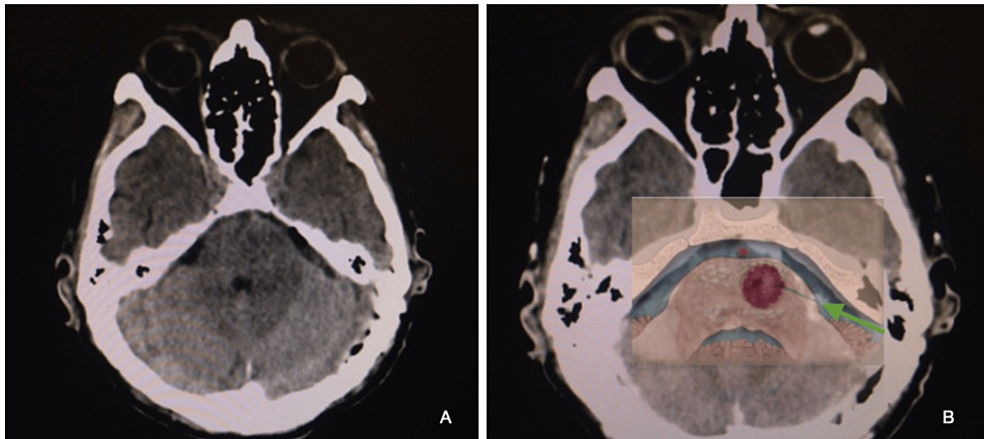
Routine postoperative head CT and illustration of pontine cavernous malformation, with depiction of a left retrosigmoid approach A: immediate postoperative head CT; B: superimposed diagram, showing the trajectory of a left-side retrosigmoid approach to an intrapontine lesion

**Figure 6:**
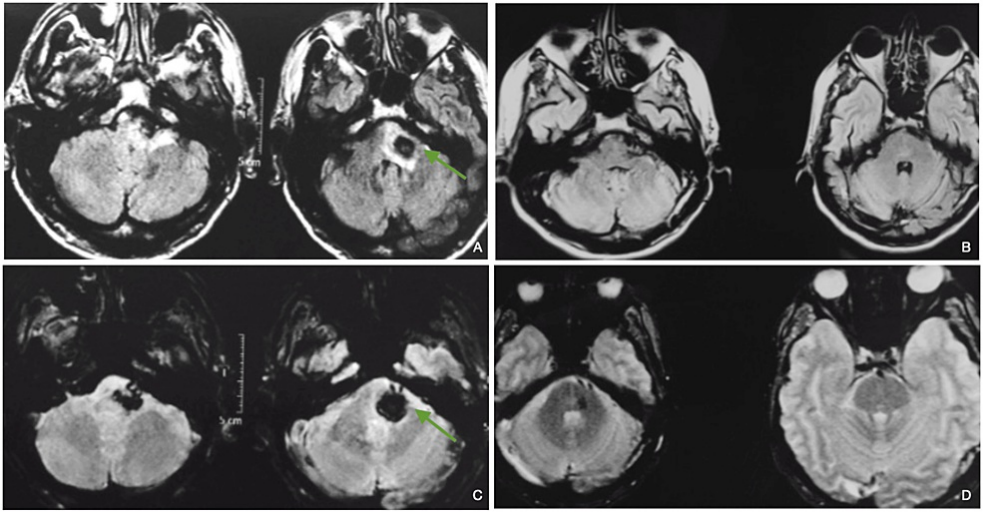
Pre and postoperative brain MRI routine delayed follow-up A and C preoperative T2WI and SWI, respectively, show a left intrapontine lesion consistent with ruptured cavernous malformation; B and D one-year follow-up T2WI and SWI, respectively, show satisfactory delayed postoperative control MRI. Green arrows denote the location of the intrapontine lesion and the approach trajectory in retrosigmoid access.

**Figure 7:**
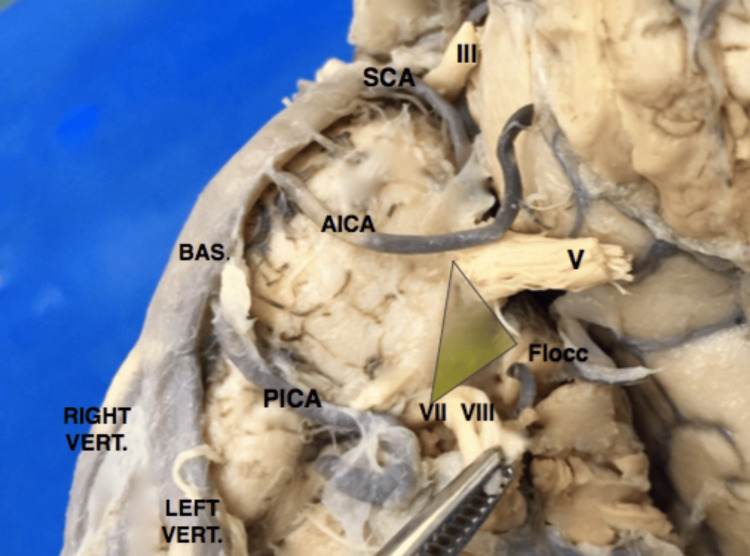
Left anterolateral pontine surface- infratrigeminal triangle Yellow shaded triangle shows the infrapontine triangle for safe entry of intrapontine lesions

The microsurgical specimens revealed that space-occupying lesions within the interior of the pons structure (Figures 1, 2, 7) could be accessed through this safe zone, demonstrating its potential to spare functional structures of the pons with greater safety. The anatomic landmarks defined by the trigeminal nerve, the flocculus of the cerebellum, and the facial-vestibulocochlear complex were consistently present in 100% of the specimens studied, indicating that these landmarks are likely to be present in most every patient requiring an approach to intrapontine lesions, which makes these landmarks reliable and reproducible for anatomical studies and, more importantly, for microsurgical interventions. Applying the microsurgical anatomy knowledge to the below-illustrated case (Figures 4, 5, 6), of a 67-year-old male patient presenting with dense right-side hemiparesis and left-side abducens and facial nerve palsy, the landmarks and safe entry zone surface area proved easy to locate and access, with minimal brainstem tissue manipulation for the best possible function preservation. Mainstay premises of brainstem cavernous malformation surgery were heedfully taken into consideration, along with the preservation of draining veins and complete lesion resection. Immediate and late postoperative follow-up images (Figures 5, 6) showed no evidence of residual or recurrent lesion, with clinical improvement of right-side hemiparesis as well as complete resolution of initially observed facial palsy, persisting only with left-side abducens paresis, which improved only partially thereafter. 

Comparison of the microsurgical anatomy of brainstem specimens (Figure 2) with intraoperative monitoring of motor tracts and cranial nerves demonstrated that an approach through the described triangle resulted in the preservation of motor tracts and cranial nerve nuclei, predicting reduced functional instability of the analyzed tracts intraoperatively and decreased risks of postoperative complications.

In summary, our findings support the existence of a safe entry zone in the lateral aspect of the pons for microsurgical approaches to intrapontine lesions. The precise measurements and consistent anatomic landmarks described in this study provide valuable guidance for neurosurgeons in accessing pontine lesions while minimizing the risk of damage to critical brainstem structures.

## Discussion

Brainstem cavernous malformations account for 9-35% of all cavernous malformations, with over 60% of them located in the pons. Managing brainstem lesions has historically posed a significant challenge in neurosurgery [6]. Thankfully, recent advances in monitoring techniques, including intraoperative neurophysiological monitoring, preoperative functional magnetic resonance (fMR), diffusion tensor imaging (DTI), and frameless stereotactic navigation, have greatly improved the success rate and feasibility of brainstem procedures [7,8]. Among these techniques, neurophysiological monitoring stands out as a critical tool. It helps neurosurgeons determine the extent of resection and allows them to adjust their surgical plans, resulting in better outcomes. Microsurgical anatomical knowledge and the description of safe entry zones, such as the one herein described, are of particular importance for patient safety. This is particularly vital in regions like the brainstem, where traditional anatomical landmarks can be distorted by tumors. Additionally, in this relatively confined area, numerous vital central nervous system pathways coexist, underscoring the importance of precise guidance [9]. Effectively managing lesions in the pontine area requires a thorough grasp of the anatomy of the posterior fossa and the morphology of the brainstem. It's crucial to have a spatial understanding of the relationships among its various components, ranging from front to back and from sides to center. This includes vital structures like the pyramidal tract, medial and lateral lemnisci, origins of cranial nerves (5th to 8th), and the longitudinal medial fasciculus [10]. It's worth noting that the most direct surgical route may not always be the safest option. For instance, when dealing with an anterolateral pons cavernous angioma, a direct approach could potentially impact the pyramidal tracts [6,11].

Various safe entry zones have been described in detail to achieve lesion removal without causing further damage [1,6,7]. The pons can be divided into several zones, such as peritrigeminal, supratrigeminal, lateral pontine zone, supracollicular, infracollicular, and the zone of the median sulcus of the fourth ventricle [7]. Access corridors such as the ones described have been reported as effective and safe for managing intrapontine lesions. Studies of microsurgical anatomy have also identified distances between critical structures [1,6]. For instance, the distance between the fifth cranial nerve and the pyramidal tract is approximately 3.1 to 5.7mm, and the dissection can be performed up to 9.5 to 13.1mm deeper, reaching the nucleus of the fifth cranial nerve in the peritrigeminal area [5,12]. Additionally, the distance from the trigeminal nerve's exit zone to the corticospinal tract averages around 9mm [13].

Since 1982, the ventrolateral portion of the pons, between the emergence of the 5th and 7th cranial nerves, has been recognized as an ideal biopsy site with minimal postoperative deficits [14]. More recently, the lateral infratrigeminal transpontine window has been explored as a low-risk entry zone. It involves incising the pons in the infratrigeminal area, between the trigeminal nerve and VII-VIII cranial nerve complex, where an avascular and relatively safe surface for incision is consistently present [6,15].

Furthermore, herein, the trigone between the trigeminal nerve, the flocculus of the cerebellum, and the facial-vestibulocochlear complex has been described for the first time in the literature. It represents another secure and effective option for intricate brainstem lesions, given the potential ease of locating these three landmarks in complex microsurgical anatomy. The choice between a transverse or a vertical incision remains a topic of discussion. Some advocate for a parallel horizontal myelotomy to preserve transverse pontine fibers better, while others prefer a vertical incision with special care regarding the intrapontine segments of the 6th and 7th cranial nerves and the corticospinal tract [12,16]. The authors prefer performing a longitudinal, cranio-caudal, corticectomy, entering through the described trigone between the trigeminal nerve exit point, the vestibulocochlear complex inferiorly, and the flocculus of the cerebellum posteriorly, in order to maintain only the appointed surface as the region for manipulation of the superficial structures and to disrupt as little of the normal anatomy as possible.

In addition to conventional approaches, endoscopic endonasal surgery has proven effective for midline exophytic pontine lesions. This approach allows access to the peritrigeminal safe zone, which is medially limited by the corticospinal tract, with average lateral and inter-pyramidal distances of 4.8mm and 3.6mm, respectively [17].

Many studies [18-23] evaluating the best approach for brainstem lesions are conducted in patients with cavernous malformations. Walter Dandy was the first acknowledged neurosurgeon to describe and operate on a brainstem cavernoma in 1928 [18]. Different approaches are used based on the relationship between the cavernous malformation and the pial surface of the brainstem [19-23]. For instance, a standard retrosigmoid craniectomy and incision of the anterolateral surface of the pons, lateral to the projection of corticospinal tracts and medial to the trigeminal nerve, have been proven to be a safe entry zone [24]. Other approaches, such as the extended retrosigmoid and translabyrinthine approaches, may also be necessary for specific cases [25,26]. The lateral transpeduncular approach is another modification of the usual retrosigmoid and posterolateral brainstem approach that results in less morbidity and mortality [27]. The suprafloccular approach, using a retrosigmoid craniectomy, provides wide exposure of the entry zones adjacent to the trigeminal nerve and the middle cerebellar peduncle and is useful for upper cerebellopontine angle regions [28,29]. These distinct approaches are more or less variations of the infratrigeminal suprafloccular approach here described but carry less detail in entering a surface area that may result in more postoperative complications, especially in regards to the pontine nucleus or corticospinal tracts.

The management of brainstem lesions has significantly improved with the aid of advanced monitoring techniques and a better understanding of the microsurgical anatomy of the region. Safe entry zones and various surgical approaches have been identified and refined, enabling neurosurgeons to effectively address brainstem lesions while minimizing damage to critical structures. These advancements have particularly benefited patients with cavernous malformations, which are relatively common in the brainstem.

Limitations 

The present study's contributions regarding brainstem cavernous malformation, microsurgical anatomy, and safe entry zones are valuable; however, a number of limitations warrant consideration. Notably, the absence of a control group for intraoperative monitoring comparison diminishes the study's capacity to definitively assess the efficacy of the safe entry zone approach and its potential correlation with surgical complications in comparison to other approaches, although that would imply ethical concerns that may preclude such a study. In addition, other considerations include the possibility of a larger anatomical specimen pool for comprehensive analysis and the comparison of the safe entry zone approach with alternative pontine surgical strategies. To enhance the study's robustness, future research designs could incorporate a control group, explore a broader array of anatomical specimens, and encompass a comparison of various pontine surgical approaches. Furthermore, a prospective study design combining microsurgical resection with the detailed identification of intrapontine structures involved in approaching such lesions would provide a more comprehensive understanding of the surgical landscape.

## Conclusions

Managing intrapontine lesions poses a significant neurosurgical challenge, given the potential catastrophic consequences of manipulating brainstem structures. Our study has, therefore, successfully identified and characterized a presumed safe entry zone that provides a surgical corridor for biopsy or microsurgical resection of such lesions. The lateral pontine triangle, delimited by the trigeminal nerve, the flocculus of the cerebellum, and the vestibulo-cochlear complex, seems to be a safe, reliable, and consistent surface area for accessing the profound structures of the pons and surgically removing lesions within. By utilizing this approach, the aim is to minimize morbidity in what was once considered an impenetrable region.

## References

[1] Cavalcanti DD, Preul MC, Kalani MY, Spetzler RF (2016). Microsurgical anatomy of safe entry zones to the brainstem. J Neurosurg.

[2] Beynon C, Kiening KL (2014). Stereotactic biopsy of brainstem lesions: A 'golden standard' for establishing the diagnosis. J Neurosci Rural Pract.

[3] Reyes-Botero G, Mokhtari K, Martin-Duverneuil N, Delattre JY, Laigle-Donadey F (2012). Adult brainstem gliomas. Oncologist.

[4] Porter RW, Detwiler PW, Spetzler RF, Lawton MT, Baskin JJ, Derksen PT, Zabramski JM (1999). Cavernous malformations of the brainstem: experience with 100 patients. J Neurosurg.

[5] Recalde RJ, Figueiredo EG, de Oliveira E (2008). Microsurgical anatomy of the safe entry zones on the anterolateral brainstem related to surgical approaches to cavernous malformations. Neurosurgery.

[6] Benner D, Hendricks BK, Benet A (2023). A system of anatomical triangles defining dissection routes to brainstem cavernous malformations: definitions and application to a cohort of 183 patients. J Neurosurg.

[7] Sala F, Manganotti P, Tramontano V, Bricolo A, Gerosa M (2007). Monitoring of motor pathways during brain stem surgery: what we have achieved and what we still miss?. Neurophysiol Clin.

[8] Deletis V, Fernández-Conejero I (2016). Intraoperative monitoring and mapping of the functional integrity of the brainstem. J Clin Neurol.

[9] Karakis I (2013). Brainstem mapping. J Clin Neurophysiol.

[10] Párraga RG, Possatti LL, Alves RV, Ribas GC, Türe U, de Oliveira E (2016). Microsurgical anatomy and internal architecture of the brainstem in 3D images: surgical considerations. J Neurosurg.

[11] Garrett M, Spetzler RF (2009). Surgical treatment of brainstem cavernous malformations. Surg Neurol.

[12] Ogut E, Armagan K, Barut C (2021). Reappraisal of the types of trigeminal porus and importance in surgical applications. Surg Radiol Anat.

[13] Yagmurlu K, Rhoton AL Jr, Tanriover N, Bennett JA (2014). Three-dimensional microsurgical anatomy and the safe entry zones of the brainstem. Neurosurgery.

[14] Baghai P, Vries JK, Bechtel PC (1982). Retromastoid approach for biopsy of brain stem tumors. Neurosurgery.

[15] Ferroli P, Schiariti M, Cordella R (2015). The lateral infratrigeminal transpontine window to deep pontine lesions. J Neurosurg.

[16] Symon L, Jackowski A, Bills D (1991). Surgical treatment of pontomedullary cavernomas. Br J Neurosurg.

[17] Essayed WI, Singh H, Lapadula G, Almodovar-Mercado GJ, Anand VK, Schwartz TH (2017). Endoscopic endonasal approach to the ventral brainstem: anatomical feasibility and surgical limitations. J Neurosurg.

[18] Dandy W (1928). Venous abnormalities and angiomas of the brain. Arch Surg.

[19] Del Curling O Jr, Kelly DL Jr, Elster AD, Craven TE (1991). An analysis of the natural history of cavernous angiomas. J Neurosurg.

[20] Robinson JR Jr, Awad IA, Magdinec M, Paranandi L (1993). Factors predisposing to clinical disability in patients with cavernous malformations of the brain. Neurosurgery.

[21] Lebayon A, Lalbaug P (2004). Brain carvenomas (Article in French). Clinique.

[22] Wang CC, Liu A, Zhang JT, Sun B, Zhao YL (2003). Surgical management of brain-stem cavernous malformations: report of 137 cases. Surg Neurol.

[23] Ohue S, Fukushima T, Kumon Y, Ohnishi T, Friedman AH (2010). Surgical management of brainstem cavernomas: selection of approaches and microsurgical techniques. Neurosurg Rev.

[24] Ferroli P, Sinisi M, Franzini A, Giombini S, Solero CL, Broggi G (2005). Brainstem cavernomas: long-term results of microsurgical resection in 52 patients. Neurosurgery.

[25] Garcia RM, Ivan ME, Lawton MT (2015). Brainstem cavernous malformations: surgical results in 104 patients and a proposed grading system to predict neurological outcomes. Neurosurgery.

[26] Cantore G, Missori P, Santoro A (1999). Cavernous angiomas of the brain stem: intra-axial anatomical pitfalls and surgical strategies. Surg Neurol.

[27] Hebb MO, Spetzler RF (2010). Lateral transpeduncular approach to intrinsic lesions of the rostral pons. Neurosurgery.

[28] Patra A, Kaur H, Chaudhary P, Asghar A, Singal A (2021). Morphology and morphometry of human paracentral lobule: an anatomical study with its application in neurosurgery. Asian J Neurosurg.

[29] Matsushima K, Yagmurlu K, Kohno M, Rhoton AL Jr (2016). Anatomy and approaches along the cerebellar-brainstem fissures. J Neurosurg.

